# Multidisciplinary Collaborative Care for Depressive Disorder in the Occupational Health Setting: design of a randomised controlled trial and cost-effectiveness study

**DOI:** 10.1186/1472-6963-8-99

**Published:** 2008-05-05

**Authors:** Moniek C Vlasveld, Johannes R Anema, Aartjan TF Beekman, Willem van Mechelen, Rob Hoedeman, Harm WJ van Marwijk, Frans F Rutten, Leona Hakkaart-van Roijen, Christina M van der Feltz-Cornelis

**Affiliations:** 1 Netherlands Institute of Mental Health and Addiction (Trimbos-institute), Utrecht, The Netherlands; 2 EMGO Institute, VU University Medical Centre, Amsterdam, The Netherlands; 3 Department of Public and Occupational Health, VU University Medical Centre, The Netherlands; 4 Body@Work, Research Centre Physical Activity, Work and Health, TNO-VU, Amsterdam, The Netherlands; 5 Research Centre for Insurance Medicine AMC-UWV-VU University Medical Centre, Amsterdam, The Netherlands; 6 Department of Psychiatry, VU University Medical Centre, Amsterdam, The Netherlands; 7 ArboNed Utrecht, The Netherlands; 8 University Medical Centre Groningen, University of Groningen, The Netherlands; 9 Department of General Practice, VU University Medical Centre, Amsterdam, The Netherlands; 10 institute for Medical Technology Assessment, Erasmus University, Rotterdam, The Netherlands

## Abstract

**Background:**

Major depressive disorder (MDD) has major consequences for both patients and society, particularly in terms of needlessly long sick leave and reduced functioning. Although evidence-based treatments for MDD are available, they show disappointing results when implemented in daily practice. A focus on work is also lacking in the treatment of depressive disorder as well as communication of general practitioners (GPs) and other health care professionals with occupational physicians (OPs). The OP may play a more important role in the recovery of patients with MDD. Purpose of the present study is to tackle these obstacles by applying a collaborative care model, which has proven to be effective in the USA, with a focus on return to work (RTW). From a societal perspective, the (cost)effectiveness of this collaborative care treatment, as a way of transmural care, will be evaluated in depressed patients on sick leave in the occupational health setting.

**Methods/Design:**

A randomised controlled trial in which the treatment of MDD in the occupational health setting will be evaluated in the Netherlands. A transmural collaborative care model, including Problem Solving Treatment (PST), a workplace intervention, antidepressant medication and manual guided self-help will be compared with care as usual (CAU). 126 Patients with MDD on sick leave between 4 and 12 weeks will be included in the study. Care in the intervention group will be provided by a multidisciplinary team of a trained OP-care manager and a consultant psychiatrist. The treatment is separated from the sickness certification. Data will be collected by means of questionnaires at baseline and at 3, 6, 9 and 12 months after baseline. Primary outcome measure is reduction of depressive symptoms, secondary outcome measure is time to RTW, tertiary outcome measure is the cost effectiveness.

**Discussion:**

The high burden of MDD and the high level of sickness absence among people with MDD contribute to the relevance of this study. The intervention is an innovative approach, with trained OPs in a new role as care managers in the treatment of MDD. If this intervention proves to be cost-effective, implementation will be very relevant for individual patients as well as for society.

**Trial registration:**

ISRCTN78462860

## Background

The burden of major depressive disorder (MDD) on the level of sickness absence in the community is huge, for society as well as for individual patient MDD is therefore responsible for enormous costs, for patients, companies and society as a whole. In the global burden of disease study, MDD is even expected to be one of the top 2 leading causes of disability-adjusted life years in 2020 [1], with a lifetime prevalence of 15.4 % and a 12-month prevalence of 5.8 % for MDD [2]. Moreover, 80% of the costs of this disorder are due to production loss [3,4]. In the Netherlands, people with MDD are absent from work 8 to 9 times more often than people without the disorder [5,6]. These high prevalences and costs, in addition to the fact that MDD with its frequent relapses is considered to be a chronic disorder, contribute to the enormous implications MDD has for society. Occupational physicians (OPs) aim to play a larger role in the care for workers with depression [7,8].

### Sickness absence

Prolonged absence from work is called the major public health problem in the western world, which leads to social deprivation of patients and their families [9]. The contribution of psychiatric disorders to sickness absence has increased and accounts for more incapacity benefit claims than musculoskeletal disorders. Among psychiatric disorders, disorders such as depression and anxiety, rather than psychotic disorders, contribute most to this rising sickness absence [9]. Moreover, the adverse economic effects related to depression are underestimated when only looking at absenteeism, because besides absenteeism, MDD is also associated with persistent presenteeism (reduced at-work job performance and productivity) [10-12].

The ability to work is an important aspect of peoples quality of life [13]. For patients, prolonged absence from work increases the risk of isolation and reduces meaningful activity [14,15]. Furthermore, the patient may become anxious to return to work, doubting his own competence and fearing that co-workers will respond with resentment or pity [14]. Longer absences are associated with a reduced probability of eventual return to work and with subsequent economic and social deprivation [9,14]. Thus, considering the implications for the patient's quality of life and the huge costs incurred by sickness absence, return to work (RTW) is very important.

Current research shows that a reduction in symptoms does not automatically lead to recovery of functioning at work [12,16-18]. In order to achieve a more rapid and more lasting RTW in patients with mental disorders, a focus on functioning at work is essential [16-18].

### Current usual care for MDD

Although evidence-based treatments are available [19], in real life, there are many obstacles [20]. First, the diagnostic process is hampered by the fact that 70% of depressed subjects present with physical symptoms to their general practitioner (GP), and not depressive symptoms [21]. Second, due to the nature of their condition, patients with MDD are less willing to accept their diagnosis, and third, do not adhere to treatment recommendations [19]. Fourth, effective methods of treatment are applied insufficiently [19,20], and care-providers do not adhere to evidence-based treatment algorithms and fifth, there is a lack of active monitoring [20,22].

Next to the insufficient implementation of the treatment of MDD, the RTW strategies of workers sick listed due to mental health problems are sub-optimal. There is a lack of communication and collaboration by Dutch OPs and GPs in the medical diagnosis and management of these employees. Dutch GPs and OPs differ in their medical diagnoses and medical management of these employees [23]. Also, GPs have a more advisory role and may pay insufficient attention to working conditions and work related interventions. This is a consequence of the fact that Dutch GPs, unlike GPs abroad, do not certify sickness absence and therefore are not obliged to pay attention to work aspects [23-25]. Lack of coordination and sub-optimal care and particularly lack of active monitoring by a care manager hampers the recovery towards functioning and RTW and leads to long-term absenteeism with unnecessarily high costs for subjects and society [26].

### Collaborative Care

Many single treatment modes for MDD have proved to be efficacious in the controlled research setting. However, they show disappointing results when implemented in daily practice. Therefore, more complex or more powerful methods of treatment are needed, adapted to the individual patient and accompanied by improvement in adherence [27,28]. In the USA, the collaborative care model turned out to be an effective answer to this problem [29-32]. In their meta-analysis, Gilbody et al. [33] confirmed the effectiveness of collaborative care in improving outcomes in depression. They reported the need for studies aimed at clarifying how collaborative care can be implemented best in European health care systems.

One of the hallmarks of collaborative care, broadly defined by Bower et al. [34] as a multifaceted organisational intervention, is the introduction of a new role, the case- or care manager. We intend to introduce this care manager in the occupational setting. The care manager coordinates care and assists in the management of patients with depressive disorders [34]. Also, in collaborative care there is collaboration between different health care professionals, such as GPs, psychiatrists and care managers. Collaborative care not only encompasses collaboration between health care professionals, as a way of transmural collaboration, but also between doctor and patient, in that the active participation of the patient is characteristic for collaborative care [30,35]. In addition, in collaborative care the progress of individual patients is continuously evaluated [34]. The organizational aspects of collaborative care are probably partly the mediating factors in the effect of this model [36].

### The intervention

In the present study, the collaborative care model is applied in the occupational health setting in the Netherlands. We intend to improve attention for work issues in the care/curative sector as well as the lack of communication in the management of MDD by using a collaborative care model with a focus on RTW.

Given that sickness absence is not only due to the personal characteristics of the patient, but is also a result of interaction with the environment (such as the workplace and the health care system), these factors should also been taken into account in the intervention [37]. For that reason, in the present study a workplace intervention (aimed at the workplace) will be combined with interventions aimed at the individual (PST, medication, self help) in a transmural care model (aimed at the health care system).

## Methods/Design

### Objectives

Primary aim of this randomised controlled trial (RCT) is to evaluate the effectiveness of collaborative care versus care as usual (CAU) in terms of severity of depressive symptoms in the treatment of MDD in the occupational health care setting. Secondary aim is to evaluate the effectiveness of the intervention in terms of RTW. Third, the cost-effectiveness will be evaluated from a societal perspective, including direct and indirect costs.

### Study design

**Figure 1:**
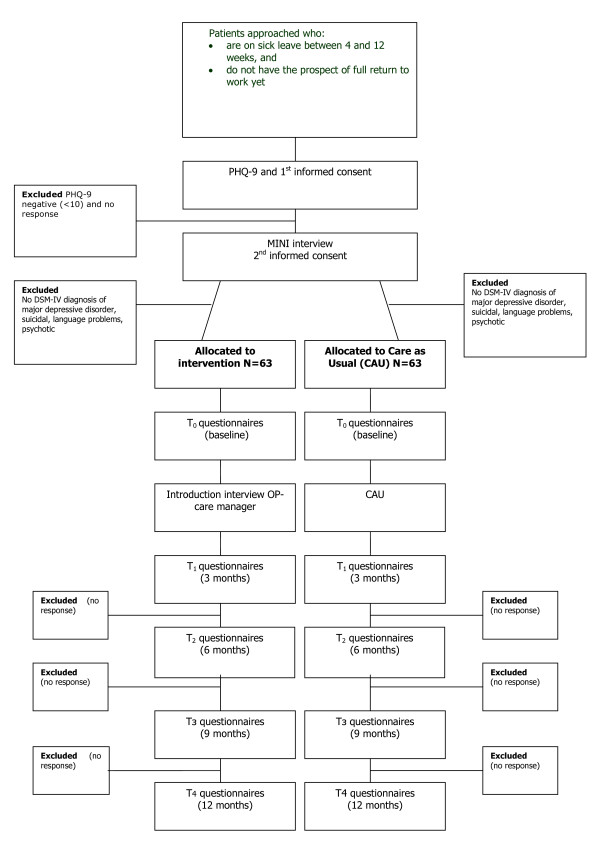
**Flowchart of the participants.** PHQ-9: Depression scale of the Patiënt Health Questionnaire, MINI: MINI-International Neuropsychiatric Interview.

This is a RCT in which collaborative care treatment for MDD will be compared to CAU in the occupational health setting. Randomisation will be at patient level. Patients allocated to the intervention group will be referred to the OP care manager in order to receive multidisciplinary treatment based on the collaborative care framework. For their sick leave, they will receive the usual care from their company's OP. Patients allocated to the usual care group will not be referred to the OP care manager and only receive sickness certification by their regular OP as CAU (see Figure 1 for a flowchart of the participants). The intervention cannot be blinded, because the patients will be aware of the allocation to either the intervention group or the usual care group. Nevertheless, all patient data will be obtained from self-report questionnaires, in order to exclude the possibility of interviewer bias. Since the OP-care managers will have patients in the intervention group only, it is not expected that they will influence the outcome of the intervention in the usual care group, so that contamination will not occur.

### Recruitment of OPs

OP-care managers will be recruited in collaboration with ArboNed, a large occupational health care service in the Netherlands. The OP-care managers will receive training in care management (including PST and the workplace intervention) before they can occupy the role of care manager in the collaborative care intervention.

### Recruitment of patients

This study will focus on employees who have been on sick leave for between 4 and 12 weeks. By choosing this duration of sick leave to intervene in, we try to prevent a transition to long-term absenteeism. Research on low back pain (LBP) suggests that treatment at a sub-acute phase (4–12 weeks) is more effective at preventing chronic disability than attempts to treat it when it has already become chronic. "The longer a worker is off work with LBP, the lower their chances of ever returning to work" [38]. In the present study we assume a comparable 'window of opportunity' for MDD and therefore choose the abovementioned duration of sick leave. The restriction of a minimum of 4 weeks of sick leave is chosen, to avoid including too many patients with spontaneous recovery.

The present study does not focus on burn-out or the broad concept of distress, which both are common health problems in working populations, but on MDD in particular. Symptoms of distress are fatigue, apathy, irritability, tension, concentration problems and insomnia [39,40]., which are also common symptoms in MDD. Also, burnout and MDD can overlap, for instance the risk of MDD is greater when burn-out is severe, compared to mild or no burn-out [41]. In this study, we will distinguish burnout and distress from MDD by the presence of the two core symptoms of MDD (anhedonia and depressive thoughts), and we will diagnose MDD according to the DSM-IV criteria [42].

Only patients whose company's OP is not an OP-care manager will be invited for participation in order to avoid contamination of effect. Patients on sick leave between 4 and 12 weeks will receive written information about the study, an informed consent form and the baseline questionnaire. They are asked if they are willing to participate in the study investigating mental problems and treatment options in the occupational health setting. If they agree to participate, they will be asked to sign the informed consent form and to return it together with the completed questionnaire to the researchers. Prior to this, the patients will be sent a letter from their company OP in which the upcoming study is announced. Also it is emphasized in this letter that participation in the study is voluntary and that refusal to participate will not have any consequences for future guidance and sickness certification.

Patients who reach the cut-off score of 10 for moderate to severe MDD on the PHQ-9 will be contacted by the research assistant. The PHQ-9 is a brief, reliable instrument that can be used to detect depressive disorders and to monitor treatment response in primary care [43,44]. The research assistant will then arrange an appointment with these patients for the administration of the mini-International Neuropsychiatric Interview (MINI) by telephone for DSM-IV classification [45,46] If a patient meets the DSM-IV criteria for MDD according to the MINI, the patient will be included in the study and after a second informed consent is obtained, the patient will be randomised. The patient will be excluded from the study if MDD is not confirmed by the MINI.

### Patient exclusion criteria

Patients who are suicidal, psychotic or with a primary diagnosis of substance abuse or dependence, as assessed by the MINI interview, will be excluded from the study. Also, patients who do not have sufficient command of the Dutch language to fill in the questionnaires and patients who are pregnant will be excluded, as well as patients with a legal involvement against their employer, e.g. due to a conflict at work.

### Treatment in the intervention group

Within the collaborative care model, the intervention will contain the following elements: contracting, adherence-enhancing techniques, manual-guided self-help, Problem Solving Treatment (PST), a workplace intervention, active monitoring and, depending on patient preference, prescription of antidepressants according to a treatment algorithm. These elements of the intervention run parallel to each other. When starting with the treatment, the patient starts with PST and the manual guided self help, and some patients will also immediately want to start with antidepressant medication. The workplace intervention will be fitted in during the first weeks of the intervention. The treatment will be monitored every two weeks and, when needed, will be intensified by adding an extra 6 sessions of PST, or by adding antidepressant medication to the treatment plan or by increasing or changing the antidepressant medication. The maximum duration of the intervention will be 18 weeks. In case of non-remission, as indicated by the PHQ-9 after 18 weeks, the patient will be referred to specialised mental health care. Antidepressant medication, when part of the treatment plan, will then be handed over to the GP.

The care in the intervention group will be provided within a multidisciplinary team comprising the OP-care manager and a consultant psychiatrist. Care management according to a protocol will be provided by the OP-care manager. The OP-care manager can consult the psychiatrist if needed and receives regular group supervision with other OP-care managers by psychiatrists. The treatment process follows an algorithm and is monitored by use of a web-based tracking system.

The content of the interventions is described below:

#### a. Contracting

During the initial visit, the OP-care manager informs the patient about MDD and treatment options. The patient can choose for treatment with or without antidepressant medication. The treatment plan is then jointly formulated by the OP-care manager and the patient. During the intervention phase, the patient is asked to fill in the PHQ-9 every two weeks in order to monitor progress.

#### b. Treatment algorithm for antidepressant medication

Antidepressant medication will be included in the treatment plan if the OP-care manager and the patient consider this necessary. In that case, the OP-care manager prescribes the medication according to a treatment protocol [47]. The protocol incorporates well-defined step up criteria and methods. Progress will be measured with the PHQ9, and the results and any adverse effects will be discussed with the patient. The OP-care managers will be supervised by the consultant psychiatrist.

#### c. Manual guided self-help

During the treatment, the patients work through a self-help manual [48]. The manual is based on several existing self-help books [49-51] and focuses on behavioural activation, negative thoughts, RTW and aspects of healthy lifestyle. Willemse et al. found that primary care patients with sub-clinical depression can benefit from self help manuals for MDD [52]. In the present study, the self-help manual is part of a complete intervention package and is therefore meant as additional to the other components of the intervention.

#### d. Adherence

Patient adherence will be improved by contracting and psycho-education and by frequent follow-up appointments in which both adherence and progress will be evaluated. Provider adherence will be improved by instructions from the researchers and by using a web-based tracking system in which the treatment algorithm is incorporated [35,53].

#### e. Problem Solving Treatment

Problem Solving Treatment (PST) is a brief, structured psychological intervention that has been shown to be effective in the management of MDD and stress related disorders [54]. The problem-solving approach is based on the common observation that emotional symptoms are often induced by problems in daily life and it encourages patients to formulate practical ways of dealing with such problems. PST is client centred en focused on the here and now. Patients will be taught to use their own skills and resources to function better [54,55].

#### f. Workplace intervention

In treatment of depressive disorder so far, insufficient attention has been paid to interventions aimed at Return To Work (RTW) [56]. However, several studies confirm the importance of taking into account the work environment and the roles of stakeholders involved in the RTW process, regardless of the type of disorder [57-62]. The disability of an employee is influenced by the actions and attitudes of the employee, the employer and the OP and by interactions occurring between them. This requires a case management approach, where disability management takes place in the workplace and work adjustments will be discussed as part of the RTW process. The workplace intervention, in which the patient, the employer and the OP-care manager participate, consists of a workplace assessment and work adjustments [57]. The OP-care manager's role is that of process mediator. The employer and the patient separately point out barriers for RTW, brainstorm for possible solutions and make a plan for implementation of solutions. The workplace intervention, based on methods used in participatory ergonomics intervention [58], relies on active participation and strong commitment of both workers and employees in identifying risk factors in the workplace and in choosing the most appropriate solutions for these risks. Like PST, it is a client centred intervention. This approach was proven effective in reducing sick leave in patients with low back pain [57] and appeared to be a useful tool in the assessment of mental workload [58,63,64]. Recently, this workplace intervention is adapted for stress related mental disorders (SMDs), using an Intervention Mapping approach [65]. In the present study, a modified version of the low back pain participatory workplace intervention is developed and will be used.

### Dutch social insurance legislation

In the present study, the OP-care manager takes an active role in curative care and is part of the treatment team. Information about the treatment can only be given to the company's OP after explicit approval of the patient. In the Netherlands, treatment and sickness certification are separated since the beginning of the 20th century. The lack of attention to working conditions by the curative sector and the lack of communication and agreement between OPs and the curative sector are consequences of this separation [7,66]. Currently, there are however several studies on their way to improve transmural occupational care and to give the OP a more prominent position in primary care [67,68]. In accordance with the separation in the Dutch legislation, the treatment of MDD and the certification in sickness absence are separated in this study. The company's OP remains responsible for the certification of sickness absence and does not take part in the treatment team. Communication between the company's OP and the treating health professionals (including the OP-care manager) in this study follows existing Dutch laws and guidelines [69,70].

### Training and treatment integrity

Prior to the start of the intervention, the participating OP-care managers receive training in collaborative care, care management, PST and the workplace intervention. The training is given by the researchers, who have previously received training from the IMPACT research group in Seattle [71] the developers of the collaborative care model. Furthermore, during the intervention the adherence of the OP-care managers to the intervention will be checked and, with the consent of the patient, audio-tapes of PST sessions will be discussed in group (peer) supervision sessions together with other OP-care managers and the PST trainer. Also, an instrument developed by Oxman et al. in order to monitor treatment integrity will be used [72].

### Treatment in the usual care group

In this study the effectiveness of the intervention is compared to usual care. Usual care is protocolled according to the OP guidelines of the Dutch Board for Occupational Medicine. As there is considerable variation in the usual care that is provided for patients with MDD, the actual care that is provided in the CAU group (e.g. medication and number of contacts with physicians) will be assessed by questionnaire.

### Data collection

Data will be collected by the Netherlands institute of mental health and addiction, in cooperation with ArboNed. Patients will be sent questionnaires and asked for their participation and written informed consent. Measurements will take place at baseline (T0), three (T1), six (T2), nine (T3) and twelve months (T4) after inclusion. The filled in questionnaires will be returned to the Netherlands Institute of Mental Health and Addiction by mail and will be processed anonymously by the researchers.

### Outcome parameters

1. The primary outcome measure is the severity of depressive symptoms, as measured according to the PHQ Depression sub-scale (PHQ9). This sub-scale is a brief and valid instrument which measures each of the DSM-IV criteria for MDD. Response is defined as a 50% reduction in symptoms [30,43,44]. Remission is defined as < 5 points on the PHQ9 [43,44].

2. The secondary outcome measure is lasting RTW, defined as the duration of sick leave due to MDD in calendar days from the day of randomization until full RTW in own or other work with equal earnings, for at least 4 weeks without (partial or full) recurrence. Also assessed will be the total numbers of days of sick leave, calculated for the entire follow up period [57,73]. Data will be derived from sick leave databases of ArboNed as well as from the Trimbos/iMTA questionnaire for Costs associated with Psychiatric Illness (TiC-P) [74].

3. The tertiary outcome measure is the cost-utility of the collaborative care intervention, compared to CAU. The cost-utility is evaluated by relating the difference in direct medical costs per patient receiving collaborative care or CAU to the difference in terms of Quality Adjusted Life Years (QALY) gained, which yields a cost per QALY estimate. We will also estimate the cost per QALY including the productivity costs. The costs will be assessed with the TiC-P, a measure commonly applied in economic evaluations of treatment in mental health care [74,75]. Quality of life will be assessed with the EuroQol (EQ-5D) [76] and the Short Form-36 (SF-36) [77], both of which are validated instruments for the measurement of general health-related quality of life. The EQ-5D descriptive system consists of five dimensions: mobility, self-care, usual activities, pain/discomfort and anxiety/depression. Each has three levels: no problems, some problems and extreme problems, thus defining a total of 243 (3^5^) distinct health states. A study that was recently carried out in the Netherlands evaluated the EQ-5D in a national setting, resulting in the 'Dutch EQ-5D tariff'. The resulting tariff is used to calculate utilities for EQ-5D health states for the cost-utility analyses of health care programmes and treatments [78,79]. Additionally, presenteeism will be assessed with the presenteeism scale of the WHO Health and Work Performance Questionnaire Short Form (HPQ Short Form) [80].

Calculating the total direct medical costs with the TiC-P, the total number of medical contacts (among which outpatient visits, length of stay in hospital, use of medication) will be multiplied by unit costs of the corresponding health care services. Reference unit prices for health care services will be applied and adjusted to the year of the study according to the consumer price index [81].

The second section of the TiC-P includes a short form of the Health and Labour questionnaire (HLQ) for collecting data on productivity losses, [82] the SF-HLQ which consists of three modules that measure productivity losses: absence from work, reduced efficiency at work and difficulties with job performance [83]. The number of days of absence from work and the actual costs of working hours missed due to health-related problems are calculated on the basis of the average value added per worker according to age and gender per day and per hour, respectively. If respondents indicate that they were absent from work during the entire recall period, data will be collected from the time when the period of long-term absence started. This additional information will be used to calculate the production losses according to the friction cost method [84,85]. The friction cost method takes into account the economic circumstances that limit the losses of productivity to society, which are related to the fact that a formerly unemployed person may replace a person who has become disabled [84].

Since the collaborative care intervention used in this study is a new intervention, a unit price per session is not known yet. To determine a reference price, a detailed cost-price study will be performed. Therefore, we will perform measurements of time for face-to-face contacts as well as indirect time per contact (e.g. consultations of other specialists) for a total of 20 sessions. Furthermore, we will estimate overhead costs based on the information of the financial department of the hospital. This will result in an estimate of the actual costs per contact. The unit cost estimate per contact will be used as a reference price per contact for the collaborative care intervention.

4. In addition, the following outcome parameters will be evaluated:

In addition to the PHQ-9, symptoms will also be assessed with the Inventory for Depressive Symptomatology Self Report (IDS-SR), measuring the severity of the symptoms of MDD as well as remission [86]. Co-morbid chronic medical illness will be measured with the CBS list, a questionnaire developed by the Dutch Central Department of Statistics. Pain will be measured according to the SF-36 Pain scale.

Patient adherence will be assessed by means of a qualitative questionnaire [53]. The treatment received in the CAU group, assessed in patients, will be measured according to the Scale Assessing Contacts between patients and practitioners [53]. The working relationship between patient and OP-care manager will be assessed by means of the Patient-Doctor Relationship Questionnaire (PDRQ-9) [87].

As a possible prognostic measure, potential work-related psychosocial factors will be assessed by the Job Content Questionnaire [88].

A process evaluation will be conducted with the first 35 cases who have been randomised in the intervention group. Both quantitative and qualitative data on the applicability, compliance, satisfaction and barriers to the protocol will be gathered. Patient satisfaction with the OP-care manager and the regular OP will be measured with the Patient Satisfaction with Occupational Health Services Questionnaire (PSOHQ) [89]. This evaluation will take place 18 weeks after randomisation, which is the maximum duration of the intervention.

### Power calculations

The primary outcome measure is response (a 50% reduction in depressive symptoms). Based on previous work [30], the expected response rate in depressive symptoms is 14,76% in the CAU group and 31,8% in the intervention group. Power calculations have been made with the usual alpha of 5% and power of 80%. In order to detect a standardized difference of 0.5 SD on the primary outcome measure, which can be considered as a clinically relevant difference, 2 × 63 patients will be needed, when taking into account two-tailed testing. An improvement of more than 5 points on the PHQ-9 can be considered as a clinically relevant difference [43].

### Analyses

#### a. Effectiveness on severity of depressive symptoms

All analyses will be performed at patient level. The data will be analysed on an intention-to-treat basis, i.e. the patients will remain in the group to which they were randomly allocated at baseline. The analyses will include t-tests, Chi-square tests and GLM Repeated measurement. The effect size will be estimated by Chi square analysis and described in Cohen's d. Possible confounders such as age, gender, immigrant status, level of education and treatment history will be included as variables in logistical regression analysis.

#### b. Effectiveness on RTW

Kaplan Meier analyses will be used to describe the association between the sick leave duration until full RTW and the group allocation. To analyse the HR of the RTW rates the Cox Proportional hazard model will be used.

#### c. Economic evaluation

The aim of the economic evaluation is to assess the cost effectiveness of collaborative care for the treatment of MDD in the occupational health setting. A cost utility analysis will be applied, the results of which will be expressed as a cost per QALY. The economic evaluation will be made from a societal perspective. Therefore, all relevant effects and costs due to resource utilisation within the healthcare system (direct medical costs) and costs due to production losses (productivity costs) will be included.

If there is missing data on costs and/or effects, and the additional uncertainty it introduces, multiple imputation will be used [90], and the Monte Carlo Markov Chain (MCMC) approach will be used to impute the missing values. The uncertainty will be assessed using bootstrapping, and the results will be presented in acceptability curves [91].

For the economic evaluation, the effects will be measured according to utility scores. In addition to the clinical outcome parameters, utility scores will supply additional information about the impact of collaborative care treatment for MDD compared to CAU on the general health-related quality of life. Furthermore, the results may be compared to a broad range of other health care interventions, also outside the field of mental health care.

### Time-frame of the study

The duration of the entire study will be four years. The preparatory period is 1 year. Subsequent to the approval of the Medical Ethical Committee, OP-care managers are recruited in collaboration with ArboNed and are trained by the investigators. The inclusion phase will last 1,5 year, and the follow-up moments will be 3, 6, 9 and 12 months after inclusion, therefore the total intervention phase will last 2,5 years. Data-analyses will take 6 months.

### Ethical principles

The study has been designed and will be carried out in accordance with the principles laid down in the Helsinki declaration (Edinburgh, Scotland amendment, October 2000). Participation in the study is voluntary. Written informed consent will be obtained from all patients and the patients will be explicitly informed about the fact that they can withdraw their consent to participate at any time, without any specific reason and with no negative consequences with regard to their future medical treatment. Patients who wish to withdraw from the study will continue to receive CAU. In addition, patients have the opportunity to consult an objective expert who is not involved in the study.

Patient names and other confidential information will be treated according to the medical confidentiality rules, and data will be separated from patient names. Each participant will be identified in the database by a number and a code, and these codes are only available to the participating investigators. Furthermore, data related to the study are stored on a protected server of the Netherlands institute of mental health and addiction, which can only be accessed by the members of the research team. The study protocol has been approved by the Medical Ethical Committee of the VU Medical Centre at August the third, 2007.

## Discussion

The high prevalence and burden of MDD, the high level of sickness absence among people with MDD and the negative consequences of prolonged sickness absence for patients as well as society contribute to the relevance of this study. Purpose of this study is to not only reduce depressive symptoms, but also to achieve an earlier, long-lasting RTW.

### Comparison with other studies

Currently, collaborative care treatment for patients with MDD is also being studied in the Netherlands in the primary care setting and the general hospital setting [92,93]. Transmural occupational care is currently being applied in the Netherlands in the RCT of Lambeek et al. [94], in which OP-care managers are responsible for the planning and coordination of care in the treatment of patients with chronic low back pain. In the RCT of van der Feltz et al., collaboration between OPs and psychiatrists is applied by offering psychiatric consultation to OPs in case of patients with common mental disorders [68].

### Strengths and limitations

Innovative of this study is the new role of the OP as the care manager in the treatment of MDD. A model for transmural care, the collaborative care model, is applied with a focus on RTW within the occupational health setting. Because of the separation between treatment and sickness certification in the Netherlands, patients are probably not much used to this, neither are the OPs themselves. However, certification in sickness absence and treatment will be provided by different OPs: the company OP and the OP-care manager. Training and close supervision will be provided to the OP-care managers and the OP-care managers will discuss their role with the patients. Also, the supervision and consultation of the psychiatrist and the web-based tracking system will facilitate working with this new model. Limitation of this study might be that in other studies effects are often found on RTW and not at symptom-level [16-18]. while our primary outcome measure is the severity of depressive symptoms and RTW is our secondary. Another limitation of this study may be that, with this study design, we will not be able to make inferences about the effectiveness of the respective ingredients of the collaborative care model (such as PST or the workplace intervention), but only about the (cost)effectiveness of the collaborative care model itself.

### Policy implications

If the collaborative care intervention proves to be cost-effective in the occupational health setting in the treatment of MDD, wider implementation may well be feasible. Since the costs of MDD are for the most part due to production loss [3,4]., implementation could be very relevant not only for individual patients and employers but also for the entire society. However, the fact that in Dutch social insurance legislation treatment and sickness certification are separated and countries differ in their level of occupational health coverage [24], might limit generalization to other countries.

## Abbreviations

MDD: major depressive disorder; GP: general practitioner, OP: occupational physician; RTW: return to work; PST: Problem Solving Treatment; CAU: care as usual; LBP: low back pain.

## Competing interests

The authors declare that they have no competing interests.

## Authors' contributions

CFC is the principle investigator, she participated in the design of the study and in writing this article. She also supervised the web based tracking system and will be available for consultation by the OP care managers. JRA participated in the design of the study, supervised the workplace intervention training and in writing the article. AB participated in the study design and in writing the article. WvM participated in the study design. LHR gave advice on the topic of cost-effectiveness and participated in writing the article. RH participated in the co-ordination of the trial and gave advice on the content of the trial. HvM supervised the PST training and gave advice in writing the article. FFR participated in development of the design of the study. MV participated in the study design, will conduct the trial and wrote the article. All participants contributed their own specific expertise and read and approved the final version of the article.

## Pre-publication history

The pre-publication history for this paper can be accessed here:


